# Editorial: Contemporary applications of machine learning and artificial intelligence for the management of heart failure

**DOI:** 10.3389/fcvm.2025.1611859

**Published:** 2025-06-03

**Authors:** Andre Rodrigues Duraes, Mansueto Gomes-Neto, Edimar Alcides Bocchi

**Affiliations:** ^1^ Federal University of Bahia (UFBA), Salvador, Brazil; ^2^ Heart Institute (InCor), São Paulo, Sao Paulo, Brazil

**Keywords:** heart failure, machine learning, artificial intelligence, biomarkers, precision medicine, telemonitoring, cardiomyopathy, healthcare technology

## Abstract

Heart failure (HF) is a complex syndrome with substantial clinical and economic impact. This editorial highlights four original articles published in *Frontiers in Cardiovascular Medicine* that showcase contemporary applications of machine learning and artificial intelligence (AI) in HF management. These studies address early diagnosis through novel biomarkers, disease stratification based on transcriptomics, mechanistic insight into apoptotic pathways, and predictive telemonitoring using real-time AI models. Collectively, these contributions exemplify the transformative potential of data-driven technologies in personalizing care and preventing decompensation in HF. We discuss both the promise and challenges of integrating these tools into routine.

**Editorial on the Research Topic**
Contemporary applications of machine learning and artificial intelligence for the management of heart failure

Heart failure (HF) remains one of the top challenges in cardiovascular medicine worldwide. It's associated with high morbidity, frequent hospitalizations, and a growing economic burden on healthcare systems. Recent advancements in precision medicine and artificial intelligence (AI) offer exciting new avenues for understanding, diagnosing, and managing HF—especially in early or pre-symptomatic stages. This issue of *Frontiers in Cardiovascular Medicine* brings together four original studies that shine light on these frontiers, from genetic biomarkers to predictive AI tools. Together, they exemplify the contemporary applications of machine learning and artificial intelligence for the management of heart failure—now emerging as key allies in advancing personalized and proactive cardiovascular care.

Yu et al. used a strong bioinformatics and machine learning approach to identify four key genes—SMOC2, OGN, FCN3, and SERPINA3—that may serve as early markers for acute decompensated heart failure (ADHF) triggered by venous congestion. Often, venous congestion sets in well before symptoms are visible. These genes showed excellent diagnostic performance (AUCs > 0.9), and the study offers a well-validated diagnostic model that could help catch worsening HF earlier. In the near future, and with the emergence of more research evaluating this complex mechanism, patients with heart failure—a condition affecting over 64 million people worldwide and associated with high rates of hospitalization and mortality—the use of a highly accurate biomarker (and that changes early) will be essential. This high precision tool will generate impact in the management of this condition. Reduction in hospitalizations, impact on the patient's social, psychological and clinical condition. Moreover, it would help decrease healthcare costs and substantially improve patients’ quality of life.

Zhang et al. identified four candidate genes (FCN3, FREM1, MNS1, and SMOC2) for diagnosing HF and went further to explore how gene expression profiles divide HF patients into subtypes. One of these groups (C3) stood out for its immune-related gene signature. Intriguingly, these same genes are linked to cancer development in broader pan-cancer datasets, suggesting some common pathways between heart failure and tumor biology. This intersection between cardiology and oncology points to new research directions in immunology and precision medicine.

Wang et al. revealed SDSL as a gene that promotes cardiomyocyte apoptosis through the PARP1 pathway. This was shown using both machine learning and in-lab validation methods such as qPCR and Western blot. SDSL's overexpression led to increased apoptosis, while silencing it reduced cellular damage. These results highlight a novel gene with strong potential as a therapeutic target—not just a diagnostic marker.

Hinrichs et al. took a very practical approach: they developed an AI model to monitor patients remotely and predict HF-related hospitalizations within seven days. Using data from the TIM-HF2 clinical trial, their model outperformed conventional rule-based systems (ROCAUC 0.855 vs. 0.727). The system could have caught 95% of upcoming hospitalizations by monitoring only the top one-third of patients with highest predicted risk. This shows how AI can streamline care and focus clinical attention where it's most urgently needed.

These studies all move in the same direction: using advanced analytics to make heart failure care more proactive, precise, and efficient. Whether it's identifying early molecular signals, characterizing patient subtypes, or predicting who's at greatest risk of hospitalization, these approaches align with the future of cardiovascular medicine.

But there are still hurdles to overcome—especially translating these tools into clinical workflows, training healthcare teams, and ensuring all patients have access to these innovations. If equity is not addressed, technological progress could risk widening existing healthcare disparities.

As guest editors, we're proud to present this Research Topic of forward-thinking research. These articles reflect the mission of *Frontiers in Cardiovascular Medicine*: to combine rigorous science with innovation and solve real-world problems. HF continues to be a major clinical challenge, but the tools to beat it are rapidly emerging.

